# Macaques Exhibit a Naturally-Occurring Depression Similar to Humans

**DOI:** 10.1038/srep09220

**Published:** 2015-03-18

**Authors:** Fan Xu, Qingyuan Wu, Liang Xie, Wei Gong, Jianguo Zhang, Peng Zheng, Qinmin Zhou, Yongjia Ji, Tao Wang, Xin Li, Liang Fang, Qi Li, Deyu Yang, Juan Li, Narayan D. Melgiri, Carol Shively, Peng Xie

**Affiliations:** 1 Department of Neurology, Yongchuan Hospital of Chongqing Medical University, Chongqing, China; 2 Institute of Neuroscience, Chongqing Medical University and the Chongqing Key Laboratory of Neurobiology, Chongqing, China; 3 Department of Neurology, the First Affiliated Hospital of Chongqing Medical University, Chongqing, China; 4 Department of Pathology, Wake Forest School of Medicine, Winston-Salem, North Carolina, USA

## Abstract

Rodent models have dominated preclinical investigations into the mechanisms of depression. However, these models-which rely on subjecting individual rodents to physical stressors - do not realistically resemble the etiopathological development of depression, which occurs naturally in a social context. A non-human primate model that better reflects the social ethological aspects of depression would be more advantageous to investigating pathophysiological mechanisms and developing antidepressant therapeutics. Here, we describe and model a naturally-occurring depressive state in a non-human primate species, the cynomolgus monkey (*Macaca fascicularis*), in a realistic social ethological context and associate the depressed behavioral phenotype with significant serum metabolic perturbations. One to two subjects per stable social colony (17–22 subjects) manifested a depressive phenotype that may be attributed to psychosocial stress. In accordance with rodent and human studies, the serum metabolic phenotype of depressed and healthy subjects significantly differed, supporting the model's face validity. However, application of the fast-acting antidepressant ketamine failed to demonstrate predictive validity. This study proposes a non-human primate depression model in a realistic social ethological context that can better approximate the psychosocial stressors underlying depression.

Depression is a debilitating psychiatric illness with a lifetime prevalence of 21% and has been shown to significantly contribute to financial distress, workplace dysfunction, increased hospitalization, and poor health status^1^,^2^,^3^. Unfortunately, the relationship between the depressive phenotype and its underlying pathophysiology remains unclear^4^. Moreover, meta-analyses of clinical trials suggest that conventional antidepressants are only marginally efficacious compared to placebos and publication biases inflate their apparent efficacy^5^. From a clinical standpoint, the current antidepressant research and development (R&D) process has been questioned; specifically, non-detection of patient subpopulations, substandard clinical trial designs, non-ideal clinical end points, lack of biomarkers, industry pressure on pharmaceutical scientists, marketing strategies, difficulty establishing public-private R&D partnerships, and publication bias in favor of positive effects have all been cited as causes for failures in the current antidepressant R&D process^6^,^7^. With these challenges in mind, developing a stronger understanding of the pathophysiology underlying depression is necessary to improve therapeutics for this debilitating mental disorder.

From a preclinical standpoint, rodent models have dominated preclinical investigations into the mechanisms of depression^8^. However, traditional rodent models, such as chronic unpredictable mild stress (CUMS), lipopolysaccharide (LPS) administration, foot-shock, the forced swimming test (FST), and tail suspension test (TST), all rely on subjecting individuals to physical stressors^9^. Thus, traditional rodent models do not realistically resemble the etiopathological development of depression, which occurs naturally in a social context^10^. Likewise, there is little or no discrimination between the state of stress and the state of depression in these models, although the two are quite easily identifiable as two different states in human beings; for example, most people do not become depressed after serious stressful experiences^11^.

Due to these limitations inherent in conventional rodent models of depression, Nestler *et al.* has commented that depression research now requires a focus on ethological models with animal subjects observed under more natural conditions^8^. Therefore, several depression research groups, including ours, have begun conducting work on more homologous non-human primate models (e.g., macaques) observed under more natural social ethological conditions^12^. Non-human primate studies have provided the best evidence for the occurrence of stress-induced social behaviors that parallel signs of depression, such as a stereotypical prostrated and socially unresponsive posture as well as exacerbation of socially submissive behaviors^13^. Interestingly, these signs have been partly reversed through chronic selective serotonin reuptake inhibitor (SSRI) administration and co-occur with cardiovascular, neuroendocrine, and neural abnormalities commonly observed in depressed patients^14^. Moreover, social ethological models of non-human primates display additional advantages in terms of understanding hierarchical dysfunction and individual psychopathology^15^,^16^.

Although ethologically valid primate models of socioaffective deficits can demonstrate strong face validity with depression, our capability to investigate their underlying circuitry is limited by technical and ethical considerations^17^. Therefore, current non-human primate depression models still require further evidence to establish model validity (e.g., ecological, etiological, face, construct, and predictive validity)^18^,^19^,^20^. In order to construct an improved non-human primate model of depression, our research group has devised a novel animal model strategy. In our initially published study, we selected a well-studied non-human primate species, the cynomolgus monkey (*Macaca fascicularis*)^21^. Next, we developed a repeatable social isolation procedure to construct a cynomolgus monkey model of depression^22^, which demonstrated that social isolation is effective in inducing depression-like behavior by significantly reducing socially dominant aggressive conflict behavior, communicative behavior, sexual behavior, and parental behavior. However, in contrast to our social isolation-induced model that assesses individual subjects isolated from their respective social groups, we have also consistently observed a subset of cynomolgus monkey subjects that display naturally-occurring depressive behavior in a social context.

To distinguish depressed macaques from healthy macaques in a social ethological context, here we assessed the behavioral changes that naturally occur in depressed cynomolgus monkeys in a colony-based social environment (i.e., the free enclosure environment). The behaviors of 20 adult female cynomolgus monkeys (*M. fascicularis*) displaying naturally-occurring depressive behavior, 10 cynomolgus monkeys displaying depressive behavior after a period of social isolation, and 20 age- and sex-matched healthy controls were recorded over an eight-day field observation period in a social ethological context. Then, the fast-acting antidepressant ketamine was applied to assess reversals in depressive behavior^23^. As depressed human patients have been shown to display significant differences from healthy individuals in blood-based metabolites^24^, serum metabolic analysis was performed to assess the metabolic differences (if any) between depressed subjects and healthy controls to further validate our model.

## Methods

### Study Site and Rearing Conditions

**Figure 1:**
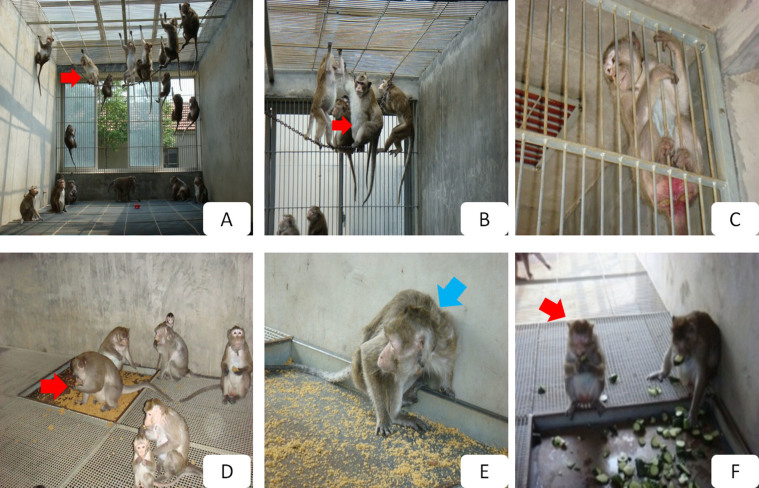
(A) Cynomolgus monkeys in a realistic social context. (B) Perching at an elevated position indicates higher dominance rank. (C) Hanging on the window or door observed with lower-ranking subjects. (D) During feeding, higher-ranking subjects take priority. (E) Lower-ranking subjects feed last in a hurried manner. (F) Higher-ranking monkeys feeding in a comfortable, seated manner. Red arrow indicates the higher-status monkey, and the blue arrow indicates the lower-status monkeys. All photographs were taken by Dr. Fan Xu.

The cynomolgus monkey feeding base at the Zhongke Experimental Animal Co., Ltd. (hereinafter “Zhongke”) is located in Suzhou, P.R.C. (E 31°07′03″ to 31°07′06″, N 120°19′08″ to 120°19′15″). Through importing cynomolgus monkeys from Guangdong and Vietnam in 1990, Zhongke established its feeding base in order to provide reliable subjects for basic experimental use and preclinical drug screening and then established a domestication and breeding base for these monkeys. Zhongke conducts regular veterinary care, daily cleanings, weekly sterilizations, and yearly inspections. Zhongke provides environmental conditions and surroundings approximating those found in the wild (Fig. 1A–F). For more information on the study site, please see our previous study^25^.

All monkeys were reared in socially-stable colonies with negligible rates of conflict, were provided with water *ad libitum*, and fed daily with fresh fruit, vegetables, and compound high-nutrition monkey food (Fig. 1D–F). To reflect wild populations they have a male: female ratio of 1: 7–11, each colony was housed in its own free enclosure composed of two males, 16–22 adult females, and their offspring of less than six months of age. There was no statistically significant difference in male: female ratios across all 52 free enclosures (*χ*^2^ = 6.2417, *df* = 51, *P* = 1.000). To better enable reproduction, the mean age in each colony was maintained near 10 ± 5 years of age.

### Subject Selection

**Figure 2:**
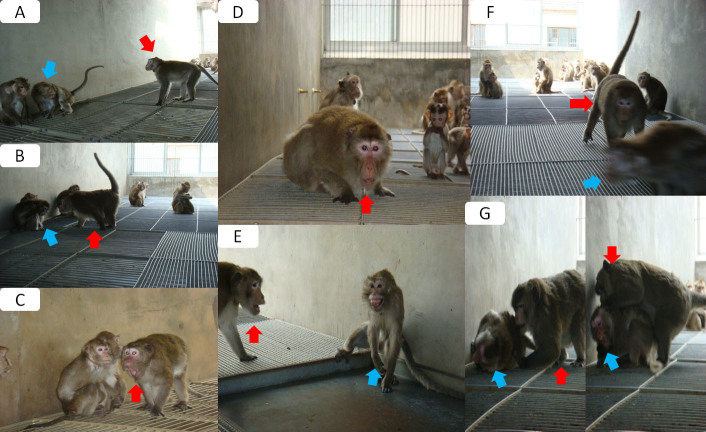
(A–G) Lower-ranking subjects facing aggression from higher-ranking subjects. Red arrow indicates the higher-status monkey, aggressing against the lower-status monkeys (indicated by blue arrow). All photographs were taken by Dr. Fan Xu.

**Figure 3:**
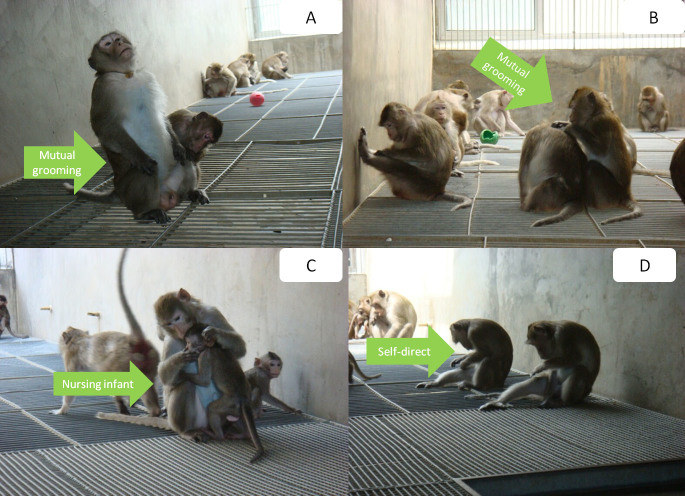
Social communication behaviors. (A, B) Grooming behavior. (C) Parental behavior. (D) Self-directed behavior. Green arrow indicates universal mutual groom behaviors. All photographs were taken by Dr. Fan Xu.

**Figure 4:**
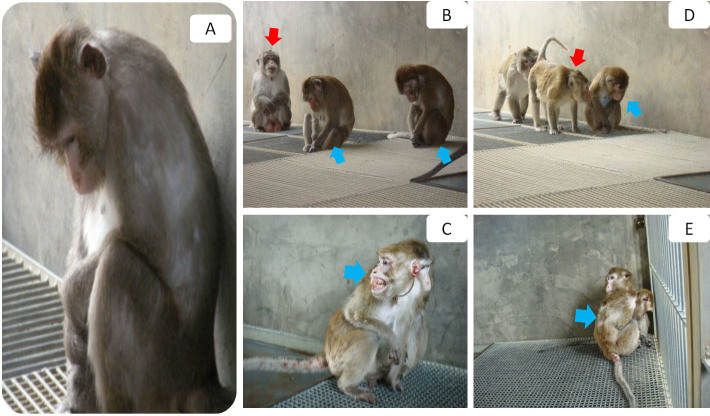
Characteristic behaviors observed in depressed subjects. (A) Slumped or collapsed posture of a depressed subject. (B) Side-by-side comparison of abnormally postured depressed subjects (right, blue arrow) and a healthy control subject (left, red arrow). (C) Post-conflict bleeding visible on the face, ear, and tail of a depressed subject. (D) Diminished social interaction with other subjects. (E) Two depressed subjects embracing in the corner of the free enclosure. All photographs were taken by Dr. Fan Xu.

From April to November 2010, by means of a scanning method on a total population of 1007 adult female cynomolgus monkeys across all 52 enclosures, we identified subjects displaying depressive behavior using the following operational definition according to Shively's criteria^26^: slumped or collapsed body posture (Fig. 1C and Fig. 2B), diminished interest in feeding and sex, and diminished communication and reciprocal grooming with others (Health Subjects, Fig. 3A–D). In total, 50 subjects met these criteria, and the frequencies of these depressive behaviors in these 50 subjects were tabulated over a two-week observational period. The 20 subjects that displayed the highest frequencies of depressive behaviors over the observational period were selected (hereinafter, naturally-occurring depressed or NOD subjects, Fig. 4A–E), and a staff veterinarian ruled out disease in these 20 subjects. To match by age and sex, 20 healthy adult female *M. fascicularis* subjects (aged 9–13 years) were selected (hereinafter, healthy controls or controls) by means of simple random sampling (random seed: 20101207) from the original pool of 1007 adult female cynomolgus monkeys. In order to provide reliable behavioral reference points for depressive behaviors in cynomolgus monkeys, 10 subjects displaying depressive behavior after a period of social isolation (hereinafter, social isolation-induced depressed or SID subjects) were also selected. Please see our previous investigation on social isolation-induced depression in the cynomolgus monkey for details.

### Experimental Procedures, Behavioral Observation, and Recording

From January 2011 to August 2013, all 40 subjects were observed for eight days by three trained observers (totaling >5000 hours of field observations). Behavioral recording methods and scored behavioral items have been described in our previous work^25^. Briefly, all behaviors of each subject were videotaped, and the three observers blindly scored all discreet behavioral items on the videotape footage using NOLDUS Observer XT software (version 10.0, Noldus Information Technology, Leesburg, PA). Data on twelve behavioral categories were gathered (Table 1) with an inter-observer reliability of greater than 85% for each discreet behavioral item.

### Ketamine Testing

The fast-acting antidepressant ketamine, a noncompetitive NMDA receptor (NMDAR) antagonist, was used to assess reversals in depressive behavior. To compare ketamine's effects in the three groups, two subjects were randomly selected from each group by simple random sampling (random seed: 20120705). Ketamine, administered at a dose of 2.5 ml/kg I.M., was injected into the distal portion of the hind limb at 9:00 A.M. We observed the six drugged subjects daily from 9:30–11:30 A.M. and 2:30–4:30 P.M. over a period of seven days.

### GC-MS Sample Preparation and Derivation

For gas chromatography-mass spectrometry (GC-MS) analysis, 15 μl of sampled serum was added to 10 μl L-leucine-13C6 (0.5 mg/ml) as an internal standard. After vortexing for 30 s, 75 μl of a methanol and chloroform mixture (methanol/chloroform: 3:1) was added. After centrifuging at 14000 rpm for 15 min, 80 μl of supernatant was evaporated to dryness under a stream of nitrogen gas. The dried residue was added to 30 μl of methoxamine hydrochloride (20 mg/ml pyridine) and incubated at 37°C for 90 min with continuous shaking. Subsequently, the solution was treated with 30 μl of BSTFA (1% TMCS) at 70°C for 60 min and placed at room temperature for 30 min.

### GC-MS Analysis

A 1 μl volume from each derived sample was injected into an Agilent 7890A/5975C GC-MS system (Agilent Technologies Inc., USA). An HP-5 MS fused silica capillary column (30 m × 0.25 mm × 0.25 μm, Agilent, USA) with a helium carrier gas flow rate of 1 ml/min was applied for metabolite separation. The injector temperature was set at 280°C. The column temperature was initially kept at 80°C for 2 min and then increased to 320°C at 10°C/min, where it was maintained for 6 min. The column effluent was introduced into the ion source of an Agilent 5975 mass selective detector (Agilent Technologies). The MS quadrupole temperature was set at 150°C, and the ion source temperature was set at 230°C. MS detection was conducted with electron impact ionization in the full scan mode (m/z, 50–600).

### Metabonomic Data Analysis

GC-MS metabolite profiles of all 40 subjects were processed after conversion into a NetCdf file format using TagFinder^27^. This processing enabled deconvolution, alignment, and data reduction to produce a list of mass and retention time pairs with corresponding intensities for all detected peaks from each data file in the data set. The resulting three-dimensional data set – including peak index (RT-m/z pair), sample names (observations), and normalized peak area percentages – were imported into SIMCA-P 11.0 (Umetrics, Umeå, Sweden) for statistical analysis.

Partial least-squares discriminant analysis (PLS-DA), a supervised multivariate approach, was performed to visually discriminate between the two experimental groups. The quality of the PLS-DA models was described by three parameters (R^2^X, R^2^Y, and Q^2^Y), which were calculated by the default leave-one-out procedure. R^2^X and R^2^Y were used to quantify the goodness-of-fit; Q^2^Y was applied to assess the predictability of the model. If the Q^2^ and R^2^ values resulting from the original model were higher than the corresponding values from the permutation test, the model was considered valid. The discriminating metabolites were obtained using a statistically significant threshold of variable influence on projection (VIP) values obtained from the OPLS-DA model (VIP > 1) and two-tailed Student's *t*-test (*P*-value < 0.05).

### Behavioral Data Analysis

Behavioral data was coded as duration (in seconds) for each discreet behavioral item per each 30-minute observational phase and presented as means ± S.D.'s. To assess the behavioral differences between NOD subjects and healthy controls and those between NOD subjects and SID subjects, Student's t-test was performed if the data was normally distributed; otherwise, the Mann-Whitney U test was applied. One-way analysis of variance (ANOVA) was performed to compare the behavioral characteristics of all three groups. In order to reduce type I errors during multiple comparisons, Bonferroni correction was used to adjust *P*-values. *P*-values of less than 0.05 were deemed significant for all analyses. All data management and statistical analysis were performed using Stata 12.0 (StataCrop LP, College Station, Texas 77845, USA).

### Ethics Statement

All animal work was conducted according to relevant national and international guidelines. In accordance with the recommendations of the Weatherall report, “The use of non-human primates in research,” the following statement has been included to document the details of animal welfare and the steps taken to ameliorate suffering in all work involving non-human primates. This study was performed in strict accordance with the recommendations in the “Guide for the Care and Use of Laboratory Animals” of the Institute of Neuroscience at Chongqing Medical University (approval no.: 20100031). State regulators and the Committee on Ethics of Animal Experimentation at Chongqing Medical University approved the protocols prior to implementation. For more detail, please refer to our previous publication^25^.

## Results

### Behaviors of Greater Duration in Healthy Controls Relative to NOD Subjects

The behaviors of greater duration in healthy controls relative to NOD subjects fell into the following behavioral categories: ingestion, rutting and mating, resting, locomotive, amicable, and miscellaneous (Tables 1, 2). First, as to ingestion behaviors, healthy controls spent longer periods ‘drinking' during the feeding phase than NOD subjects (Fig. 1D–E). Second, with respect to resting behaviors, healthy controls spent more time resting on elevated perches (i.e., ‘perching on shelf' and ‘hanging on iron chain') than NOD subjects. Third, respecting locomotive behavior, healthy controls presented with significantly longer durations moving along elevated perches (i.e., ‘walking on the shelf' and ‘walking on the iron chain') and the floor (i.e., ‘quadrupedal walking on the floor' and ‘standing') than NOD subjects. Fourth, as to amicable behavior, healthy controls received more amicable grooming (i.e., ‘being groomed'), groomed others more (i.e., ‘grooming'), and nursed their own infants more (i.e., ‘nursing infant') relative to NOD subjects. Finally, as to miscellaneous behavior, healthy controls spent more time on one self-directed behavior (i.e., ‘scratching by foreleg') than NOD subjects.

### Behaviors of Greater Duration in NOD Subjects Relative to Healthy Controls

The behaviors of greater duration in NOD subjects relative to healthy controls fell into the following behavioral categories: ingestion, resting, locomotive, thermo-regulatory, conflict, and miscellaneous (Tables 1, 3 and Fig. 2, 3). First, as to ingestion behaviors, NOD subjects more often selected safer and more remote places to feed (i.e., ‘feeding while hanging') to avoid confrontation. Second, in terms of resting behaviors, NOD subjects selected safer and more remote areas to rest such as ‘sitting on floor.' Third, with regard to locomotive behaviors, NOD subjects were more predominantly found walking on the skylight (i.e., ‘walking on skylight') to avoid potential attack relative to healthy controls. Fourth, with respect to thermo-regulatory behavior, NOD subjects spent more time on solitary huddling or embracing a conspecific (i.e., ‘huddling'/‘embracing'). Finally, as to miscellaneous behaviors, NOD subjects spent more time on two particular self-directed behaviors (i.e., ‘scratching by hind leg' and ‘licking tail') than healthy controls.

### Behavioral Similarities between NOD and SID Subjects

We compared the behaviors of the subjects from the two models of depression, NOD and SID, to determine their similarities, which fell into the following behavioral categories: ingestion, rutting and mating, resting, amicable, conflict, vigilance, and locomotive (Tables 1, 4 and Fig. 4). As to ingestion behavior, both NOD and SID subjects shared similar levels of feeding after others had finished feeding (i.e., ‘licking residue from floor'), chewing food (i.e., ‘chewing'), and feeding from a perched position (i.e., ‘feeding while perched'). As to rutting and mating behavior, both NOD and SID subjects shared similar levels of sexual intercourse (i.e., ‘copulating'). As to resting behaviors, both NOD and SID subjects shared similar levels of resting in safer and more remote places (i.e., ‘hanging on iron chain,' ‘hanging on skylight,' and ‘hanging on ventilator') in addition to other resting behaviors (i.e., ‘sitting on floor,' ‘perching on shelf,' ‘lying on floor,' ‘lying on the board,' and ‘sitting and sleeping'). As to amicable behavior, both NOD and SID subjects shared similar levels of grooming others (i.e., ‘grooming'). As to conflict behavior, both NOD and SID subjects shared similar levels of conflict with others (i.e., ‘driving,' ‘attacking,' ‘fleeing,' ‘pulling foreleg,' ‘biting,' and ‘being attacked'), which may be related to their shared levels of vigilance behavior (i.e., ‘watching company' and ‘miscellaneous calling'). In regard to locomotive behavior, both NOD and SID subjects shared similar levels of moving in safer and more remote areas (i.e., ‘climbing') and walking on the floor (i.e., ‘quadrupedal walking on floor').

### Behavioral Differences between NOD and SID Subjects

We also compared the behaviors of the subjects from the two models of depression, NOD and SID, to determine their differences (Table 5). SID subjects spent almost six times more time than NOD subjects ‘drinking'. SID subjects also spent more time than NOD subjects on certain self-directed behaviors (i.e., ‘rubbing paw on floor'). SID subjects also spent more time than NOD subjects ‘mounting,' ‘being groomed,' and ‘standing.'

In contrast, NOD subjects spent more time than SID subjects resting (i.e., ‘hanging on window or door'). NOD subjects also spent more time than SID subjects on maternal-infant care (i.e., ‘nursing infant' and ‘holding infant'). NOD subjects also spent more time than SID subjects receiving aggression from others (i.e., ‘being threatened'), which may have contributed to increased submissive behavior (i.e., ‘presenting buttocks').

### Behavioral Differences among Control, NOD, and SID Subjects

In order to better define the reliable behavioral aspects of NOD subjects, the behavioral presentations of all three groups were compared (Table 6). Notably, according to the post hoc test, both NOD and SID subjects presented similar behavioral characteristics that were significantly different from the control group, including ‘sitting on floor,' ‘hanging on iron chain,' ‘nursing infant,' ‘walking on shelf,' and ‘walking on iron chain.' First, with regard to first two actions in the resting category, both NOD and SID subjects spent more time ‘sitting on floor' and ‘hanging on iron chain' relative to controls. Second, both NOD and SID subjects spent less time ‘nursing infant' than controls. Third, both NOD and SID subjects spent less time ‘walking on shelf' and ‘walking on iron chain' relative to controls.

### Behavioral Effects of Ketamine Administration

In order to assess prediction validity, the fast-acting anti-depressant ketamine was used to comparatively assess reversals in depressive behavior in NOD and SID subjects relative to healthy controls. With regard to the duration of behaviors, both NOD and SID subjects displayed decreased durations of ‘chewing' and increased durations of ‘hanging on window or door' post-ketamine administration with no such changes observed in healthy controls. With respect to the frequency of behaviors, both NOD and SID subjects displayed decreased frequencies in ‘feeding while sitting' and ‘chewing' post-ketamine administration with no such changes observed in healthy controls (Table 7).

Three behaviors were reduced across all three groups post-ketamine administration. The duration of ‘feeding while perched' was completely eliminated in healthy controls, was reduced by a factor of 0.64 in NOD subjects, and was reduced by a factor of 0.018 in SID subjects. The frequencies of ‘watching company' were reduced by factors of 0.77, 0.81, and 0.64 in healthy controls, NOD subjects, and SID subjects, respectively. The frequencies of ‘scratching by foreleg' were reduced by factors of 0.61, 0.73, and 0.44, respectively.

### Serum Metabonomic Analysis

PLS-DA analysis was carried out to explore the metabolic differences between NOD subjects and healthy controls. The resulting score plots of the PLS-DA model revealed that NOD subjects were significantly distinguishable from healthy controls (R^2^X = 0.802, R^2^Y = 0.936, Q^2^ = 0.444). The values of all three parameters (R^2^X, R^2^Y, and Q^2^Y) were positive, demonstrating a robust metabolic difference between NOD subjects and healthy controls.

To identify the differential metabolites responsible for the discrimination between NOD subjects and healthy controls, the corresponding loading plots of the PLS-DA model were analyzed. This analysis resulted in the identification of 27 differential metabolites with a value of VIP > 1 and *p* < 0.05 (Table 8). As compared to healthy controls, NOD subjects were characterized by higher levels of silanamine, benzamide, sedoheptulose, and glycerol, in addition to lower levels of urea, leucine, serine, threonine, butanoic acid, threitol, methionine, proline, phenylalanine, asparagine, monoamidomalonic acid, 2-keto-gluconic acid, arabinofuranose, phosphoric acid, ornithine, citric acid, 1,4-butanediamine, fructose, glucose, inositol, myo-inositol, turanose, and α-D-glucopyranoside. Functional analysis of these differential metabolites demonstrated that changes in NOD subjects primarily involved disturbances in amino acid metabolism (i.e., leucine, serine, threonine, methionine, proline, phenylalanine, asparagine, and ornithine).

## Discussion

The cynomolgus monkey (*Macaca fascicularis*) model described here better resembles the etiological development of depression and contains three key elements characteristic of a naturally-occurring model of depression: (i) observation is conducted in a natural colony-based social environment; (ii) the colony's social hierarchy produces stress-inducing social competition; and (iii) the depressed disease state naturally arises with no experimental manipulation to disturb the etiological process. This study is the first to conclusively associate the depressed behavioral phenotype in the cynomolgus monkey with significant serum metabolic changes, indicating that the depressed behavioral phenotype relates to specific underlying pathophysiological changes.

Studies on other animal species have also taken a social ethological approach to investigating depression with similar findings. Carole et al. observed the behavior of 59 working horses in their domestic environment and found 24% of the horses presented with “withdrawn” behavior suggestive of a depressive syndrome (e.g., atypical posture (stretched neck), unusual gaze, fixity of head and ears)^28^. As compared to the “non-withdrawn” horses (healthy controls) from the same stable, “withdrawn” horses appeared more indifferent to environmental stimuli in their home environment and reacted more emotionally to challenging situations. Carole et al. also noted that similar apathetic behavior has been observed in other domesticated animal species living under unfavorable conditions (e.g., sows, sheep, chicks, pigs). Moreover, numerous rodent studies have investigated dominance-subordination relationships to examine the effects of social stress^29^. For example, Golden reported that C57BL/6J mice repeatedly subjected to bouts of social defeat by larger and more aggressive CD-1 mice resulted in the development of a depressive-like syndrome characterized by enduring deficits in social interactions^30^.

Although these previous animal models of depression have been valuable, an ideal depression model should increase the ease with which an analogue of depression or dysthymia may be evoked and should demonstrate five types of validity: ecological, construct, etiological, face, and predictive^18^,^19^,^20^ As discussed below, our social ethological model of depression more adequately addresses four of these five types of validity relative to traditional animal models of depression.

With respect to ecological validity (i.e., how closely our model resembles the natural environmental conditions in which the human disease state occurs)^31^, our naturally-occurring depression model is superior to traditional rodent models as it occurs in a realistic social ethological context. Similar to many modern human societies, our model possesses a number of interacting subjects that constitute a relatively stable socially-hierarchical society^32^. Notably, adult female cynomolgus monkeys were selected for this study, as they organize themselves into a stable social hierarchy in conjunction with one or two males^25^. Also similar to many modern human societies, scarce resources that form the basis of stress-inducing social competition (e.g., food, sex) are unevenly distributed among different social classes^33^. Thus, compared to traditional rodent models of depression, our macaque model more closely approximates the natural environmental conditions under which depression arises in humans.

With respect to construct validity (i.e., how closely our model resembles the proposed theoretical construct of the human disease state), several biological constructs for the development of depression have been proposed with considerable overlap between them (e.g., HPA-axis hyperactivity, the monoamine theory, the cytokine hypothesis/macrophage theory, and neurostructural change); however, in the case of all these constructs, psychosocial stress has been proposed as a bridge between the pathophysiology of depression and its predominantly psychosocial risk factors^34^. Our naturally-occurring depression model is superior to traditional rodent models in terms of construct validity, as our model includes psychosocial stressors in a realistic social ethological context (e.g., conflicts over feeding, sex, and resting areas) that are akin to the psychosocial stressors that underlie depression development in humans. Moreover, these psychosocial stressors have been physiologically validated, as previous studies in adult female cynomolgus monkeys meeting the identical selection criteria applied here have been shown to display elevated cortisol levels and HPA-axis dysfunction^26^,^35^,^36^.

With respect to etiological validity (i.e., how closely our model resembles the causative conditions of the human disease state)^37^, our naturally-occurring model of depression is superior to traditional rodent models, as our model does not rely upon direct experimental manipulation but naturally occurs in a social environment designed to approximate wild conditions in terms of colony size and male: female ratio. Accordingly, our group and Shively *et al.* have previously demonstrated that depressive behavior in captive cynomolgus monkeys is spontaneous and not induced by specific experimental manipulation^12^. In addition, similar to modern humans, our model involves chronic mild psychosocial stressors that are randomly distributed throughout daily social life that accumulate over time. As a result, psychosocial stressors naturally arise from resource-based competition in the social context of the macaque colony. Thus, our model demonstrates superior etiological validity relative to conventional rodent models by better approximating the psychosocial stressors encountered by adult humans living in a resource-based competitive society^8^. That being said, the etiology of depression is a complex, multifactorial process that involves more than just psychosocial stressors but has also been shown to involve lifestyle (e.g., diet, sleep, and exercise) and genetic factors as well^38^,^39^. Our model does not account for these other key etiological factors, a fact which reduces the etiological validity of our model.

With respect to face validity (i.e., how closely the model resembles the human disease state)^40^, NOD subjects presented with a slumped or collapsed body posture and maintained a low dominance rank in the social hierarchy as demonstrated by feeding last and selecting relatively remote and safe places to rest (Tables 2, 3). (Macaque colonies are socially hierarchical with higher-ranked subjects, such as the alpha-male and alpha-female, characterized by eating first and resting on elevated perches for observing their colony; NOD subjects spent significantly less time feeding first and resting on these elevated perches.) Moreover, NOD subjects received more aggression from other subjects and were less involved in reciprocal grooming behavior with other subjects. These findings are similar to those previously found in Shively's work on cynomolgus monkeys^26^ and resemble the abnormal posture and low-status, relational victimization associated with depressed human patients^41^,^42^,^43^. It should be noted here that juvenile monkeys were not selected for this study, as juveniles may not possess the psychological maturity to fully experience the depressive mood state and/or physically manifest the depressive phenotype and would, therefore, not qualify to support the face validity of our naturally-occurring depression model.

In further support of our model's face validity, GC-MS-based metabolomic profiling displayed significant disturbances amino acid metabolites (i.e, leucine, serine, threonine, methionine, proline, phenylalanine, asparagine, and ornithine) as well as carbohydrate and fatty acid metabolites (e.g., glucose, fructose, citric acid, glycerol) in NOD subjects, findings which are consistent with previous serum/plasma metabolomic analyses that have also observed significant abnormalities in these metabolic pathways in rodent models of depression and depressed human patients^24^,^44^,^45^. More specifically, significant decreases in three differential metabolites identified in NOD subjects here -- leucine, glucose, and myo-inositol -- were also observed in our previous NMR-based plasma metabolomic study in depressed humans^24^. However, several other amino acid, carbohydrate, and fatty acid metabolites that were found to be dysregulated in depressed human patients (e.g., valine, lactate, alanine, acetate, pyruvate, glutamine, taurine, glycine, unsaturated lipids) were not significantly affected in our NOD subjects here. Moreover, in a previous GC-MS metabolomic study in older adult patients, significant decreases in three other differential metabolites -- inositol, citrate, and glycerol – were found in the plasma of depressed older adults^46^. Specifically, although our results in NOD subjects for inositol and citrate were consistent with the previous study, glycerol was upregulated in NOD subjects here but was found to be downregulated in depressed older adults. According to Paige et al.'s commentary, these changes in citrate and glycerol in depressed older patients are suggestive of a reduction in fatty acid oxidation; consequently, these alterations were accompanied by changes in glycerate and medium-chain fatty acids (e.g., myristate, palmitate, stearate, oleate). However, we did not observe changes in glycerate or fatty acid levels in the NOD subjects here. Moreover, several differential metabolites observed in NOD subjects here (e.g., silanamine, benzamide, sedoheptulose, urea, butanoic acid, threitol, monoamidomalonic acid, 2-keto-gluconic acid, arabinofuranose, phosphoric acid, 1,4-butanediamine, turanose, and α-D-glucopyranoside) have not been previously associated with plasma/serum metabolite profiles in human depression, which may be partly attributable to inter-species differences in dietary intake, commensal microbiota composition, and metabolic processing. These combined findings suggest some significant similarities and differences between the NOD metabolic phenotype and the human depressive metabolic phenotype.

With regard to predictive validity (i.e., the model's selectivity in response to specific compounds, such as antipsychotics or antidepressants)^23^,^47^,^48^, the fast-acting anti-depressant ketamine was used to comparatively assess reversals in depressive behavior in NOD subjects relative to healthy controls. NOD subjects displayed decreased durations of ‘chewing' and increased durations of ‘hanging on window or door' and decreased frequencies in ‘feeding while sitting' and ‘chewing' post-ketamine administration with no such changes observed in healthy controls. Therefore, unfortunately, ketamine administration did not produce reversals in depressive behavior in NOD subjects. As field observations were limited to only two subjects over a course of seven days, future studies should include a larger sample set of NOD subjects treated with conventional antidepressant therapy observed over a longer duration.

There are several notable limitations to this study. First, a self-controlled study design that allowed for the direct comparisons before and after social isolation as well as before and after ketamine administration would have better controlled for individual differences. Future studies involving interventions in this species should seek to self-control to account for individual variations. Second, this study failed to provide insight as to why ketamine administration did not produce expected reversals in depressive behavior in NOD subjects; thus, future studies should apply conventional antidepressant therapy observed over a longer duration in order to gather more evidence to support the predictive validity of this model. Third, our metabolic data was captured at one specific time point, while behavioral observation was conducted over a longer period of time, making the performance of correlation/regression analyses to examine potential relationships between behavioral and metabolic measurements impossible. A future study will have to monitor both behavioral and serum metabolite changes in tandem over a period of time in order to robustly perform these analyses.

## Conclusion

Here, we describe and model a naturally-occurring depressive state in a non-human primate species, the cynomolgus monkey (*Macaca fascicularis*), in a realistic social ethological context and associated the depressed behavioral phenotype with significant serum metabolic perturbations. In accordance with rodent and human studies, the metabolic phenotype of the NOD subjects and healthy controls significantly differed. This model's stable depression phenotype demonstrates superior validity to traditional rodent models of depression by better approximating the psychosocial stressors encountered by adult humans living in a resource-based competitive society and can aid further investigation into the mechanisms underlying depression.

## Author Contributions

F.X. and P.X. conceived and designed the study and F.X. prepared the figures 1–4; F.X., X.L., L.X., Y.J., T.W., Q.W., W.G., P.Z., Q.L., J.L., J.G.Z. and L.F. conducted the field observations and Noldus analysis; F.X., D.Y.Y. and Q.Z. analyzed the data; F.X. and P.X. wrote the main manuscript; C.S. and N.M. supervised the data analysis and edited the manuscript for intellectual content and style. All authors reviewed and approved the manuscript prior to its submission.

## Figures and Tables

**Table 1:** Descriptions of Behavioral Categories and Items

Behavioral Categories∧	Behavioral Items
Ingestion	Searching, feeding while squatting, feeding while hanging, feeding while sitting, drinking, chewing, licking residue from floor, picking remaining food, feeding while perched, suckling
Thermo-regulatory	Huddling, quivering, embracing
Rutting and estrous	Licking genital area, presenting buttocks, peri-vulvar discoloration, sniffing ano-genital area, sniffing urine, rolling tongue, homosexual roaring, homosexual mounting, tail arching
Mating	Mounting, copulating, post-copulation guarding, post-copulation standing, ejaculating, masturbating
Resting	Sitting on floor, sitting on floor facing wall, perching on shelf (socially dominant), sitting on ring, lying on floor, lying on shelf, hanging on window or door (non-socially dominant), hanging on skylight, hanging on iron chain, hanging on ventilator, sitting and sleeping
Parental	Nursing infant, holding infant, defending infant, licking anus of infant, checking anus of infant
Amicable	Grooming, being groomed, embracing
Conflict	Driving (socially dominant, aggressive), attacking (socially dominant, aggressive), fleeing (non-socially dominant), pulling foreleg, pulling hind leg, protracting ears, threatening (socially dominant, aggressive), being threatened (non-socially dominant), being attacked (non-socially dominant), parallel pacing, biting (socially dominant, aggressive)
Vigilance	Shifting position, alarmed jumping, watching company, alarmed calling, miscellaneous calling, shaking cage
Locomotive	Galloping, walking on shelf (socially dominant), quadrupedal walking on floor (non-socially dominant), moving, climbing, walking on iron chain, walking on skylight, standing, stepping, trotting
Communication	Lip smacking, sniffing, voiding, alarmed calling, miscellaneous calling
Miscellaneous (self-directed)	Huddling (solitary), shaking body, playing (solitary), licking hair, scratching by hind leg, scratching by foreleg, yawning, licking hand, catching pest, digging anus, rubbing palm on floor, licking tail, shaking ID card

^∧^Behavioral items and categories established by systematic ethogram of *M. fascicularis*.

**Table 2:** Behaviors of Greater Duration in Healthy Controls Relative to NOD Subjects

Behavior	Healthy controls	NOD subjects	*P*-value	Adjusted *P*-value∧
Drinking*	6.16 ± 23.55	2.41 ± 12.34	0.0000	0.0000
Sitting on floor facing wall*	9.06 ± 49.27	3.62 ± 31.16	0.0001	0.0058
Perching on shelf*	248.95 ± 439.31	185.41 ± 412.27	0.0000	0.0000
Hanging on iron chain*	36.93 ± 160.36	10.84 ± 103.5	0.0000	0.0000
Nursing infant*	15.27 ± 66.18	6.06 ± 33.23	0.0000	0.0000
Grooming*	96.53 ± 209.36	59.76 ± 164.79	0.0000	0.0000
Being groomed*	95.79 ± 194.95	56.75 ± 133.14	0.0000	0.0000
Walking on shelf*	4.64 ± 10.92	3.60 ± 10.53	0.0000	0.0000
Quadrupedal walking on floor*	78.92 ± 72.94	68.36 ± 63.45	0.0008	0.0464
Walking on iron chain*	0.78 ± 5.49	0.06 ± 1.01	0.0000	0.0000
Walking on the skylight*	1.53 ± 6.19	3.52 ± 19.79	0.0001	0.0058
Standing*	13.69 ± 29.86	9.87 ± 24.33	0.0000	0.0000
Scratching by foreleg*	77.60 ± 131.87	56.85 ± 97.74	0.0000	0.0000

Data listed as means ± SDs.

^∧^Bonferroni correction used to reduce type I error for multiple comparisons.

*P < 0.05.

**Table 3:** Behaviors of Greater Duration in NOD Subjects Relative to Healthy Controls

Behavior	Healthy controls	NOD subjects	*P*-value	Adjusted *P*-value∧
Feeding while hanging*	2.23 ± 20.33	6.26 ± 39.03	0.0006	0.0348
Huddling/embracing*	171.18 ± 390.27	278.84 ± 474.32	0.0000	0.0000
Sitting on floor*	947.78 ± 564.24	1018.96 ± 601.74	0.0005	0.0290
Walking on skylight*	1.53 ± 6.19	3.52 ± 19.79	0.0001	0.0058
Scratching by hind leg*	2.41 ± 4.36	4.27 ± 7.32	0.0000	0.0000
Licking tail*	0.35 ± 3.12	2.46 ± 22.59	0.0004	0.0232

Data listed as means ± SDs.

^∧^Bonferroni correction used to reduce type I error for multiple comparisons.

*P < 0.05.

**Table 4:** Behavioral Similarities between NOD and SID Subjects

Behavior	NOD subjects	SID subjects
Chewing	73.7 ± 182.80	74.32 ± 188.09
Licking residue from floor	0.92 ± 11.67	0.76 ± 6.69
Feeding while perched	3.25 ± 25.87	2.38 ± 25.25
Copulating	0.49 ± 2.39	0.35 ± 1.43
Sitting on floor	1018.96 ± 601.74	1078.43 ± 546.62
Perching on shelf	185.41 ± 412.27	216.70 ± 464.13
Lying on floor	26.07 ± 111.65	33.91 ± 143.93
Lying on the board	5.76 ± 55.14	1.56 ± 12.35
Hanging on iron chain	10.84 ± 103.50	12.92 ± 78.92
Hanging on skylight	17.27 ± 122.16	3.44 ± 18.30
Sitting and sleeping	0.44 ± 8.43	0.26 ± 2.94
Hanging on ventilator	1.62 ± 27.74	0.00 ± 0.00
Grooming	59.76 ± 164.79	74.73 ± 182.76
Driving	0.09 ± 1.56	0.17 ± 1.94
Attacking	0.04 ± 1.29	0.00 ± 0.00
Fleeing	0.09 ± 1.12	0.05 ± 0.79
Pulling foreleg	0.008 ± 0.13	0.007 ± 0.11
Biting	0.02 ± 0.62	0.00 ± 0.00
Being attacked	0.38 ± 2.66	0.26 ± 2.14
Watching company	1300.15 ± 429.11	1269.42 ± 423.15
Miscellaneous calling	0.02 ± 0.50	0.00 ± 0.00
Quadrupedal walking on floor	68.36 ± 63.45	75.14 ± 72.27
Climbing	0.06 ± 1.00	0.05 ± 0.61

Data listed as means ± SDs.

Only behaviors with no significant differences between NOD and SID subjects are listed.

**Table 5:** Behavioral Differences between NOD and SID Subjects

Behavior	NOD subjects	SID subjects	*P*-value	Adjusted *P*-value∧
***SID > NOD***				
Drinking*	2.41 ± 12.34	12.93 ± 38.91	0.0001	0.0058
Mounting*	0.002 ± 0.04	0.13 ± 0.95	0.0001	0.0058
Being groomed*	56.75 ± 133.14	114.60 ± 229.09	0.0000	0.0000
Standing*	9.87 ± 24.33	16.86 ± 30.71	0.0000	0.0000
Rubbing paw on floor*	0.10 ± 1.13	1.71 ± 8.55	0.0000	0.0000
***NOD > SID***				
Presenting buttocks*	1.80 ± 14.09	0.00 ± 0.00	0.0000	0.0000
Hanging on window or door*	168.49 ± 379.64	62.69 ± 213.01	0.0003	0.0174
Nursing infant*	6.06 ± 33.22	0.34 ± 4.99	0.0000	0.0000
Holding infant*	144.43 ± 405.47	1.77 ± 8.40	0.0001	0.0058

Data listed as means ± SDs.

^∧^Bonferroni correction used to reduce type I error for multiple comparisons.

*P < 0.05.

**Table 6:** One Way-ANOVA Comparison of All Three Groups

Behavior	Controls	NOD subjects	SID subjects	F	*P*-value	Adjusted *P*-value∧
Feeding while hanging	2.23 ± 20.33	6.26 ± 39.03	1.29 ± 10.50	6.61	0.0014	0.0812
Feeding while sitting	61.67 ± 166.73	30.63 ± 88.49	58.41 ± 144.60	16.74	0.0000	0.0000
Drinking	6.16 ± 23.55	2.41 ± 12.34	12.93 ± 38.91	25.82	0.0000	0.0000
Picking remaining food	31.90 ± 131.59	15.67 ± 66.99	40.95 ± 147.47	9.10	0.0010	0.0580
Embracing with conspecifics	171.18 ± 390.28	278.83 ± 474.32	225.31 ± 445.47	18.83	0.0000	0.0000
Presenting buttocks	2.73 ± 13.65	1.80 ± 14.09	0.00 ± 0.00	4.50	0.0120	0.6960
Sitting on floor*	947.78 ± 564.24	1018.96 ± 601.74	1078.43 ± 546.62	7.41	0.0000	0.0000
Sitting on floor facing wall	9.06 ± 49.27	3.62 ± 31.16	14.52 ± 82.53	7.30	0.0007	0.0406
Perching on shelf	248.95 ± 439.31	185.41 ± 412.27	216.70 ± 464.13	6.69	0.0013	0.0754
Hanging on window or door	127.57 ± 335.53	168.49 ± 379.64	62.69 ± 213.01	10.22	0.0000	0.0000
Hanging on iron chain*	36.93 ± 160.36	10.84 ± 103.50	12.92 ± 78.92	12.66	0.0000	0.0000
Hanging on skylight	6.57 ± 69.11	17.27 ± 122.16	3.44 ± 18.30	4.71	0.0090	0.5220
Nursing infant*	15.28 ± 66.18	6.06 ± 33.23	0.34 ± 4.99	14.44	0.0000	0.0000
Holding infant	146.67 ± 380.36	144.43 ± 405.47	1.77 ± 8.40	14.83	0.0000	0.0000
Grooming	96.53 ± 209.36	59.76 ± 164.79	74.73 ± 182.76	11.66	0.0000	0.0000
Being groomed	95.79 ± 194.94	56.75 ± 133.14	114.60 ± 229.10	20.15	0.0000	0.0000
Walking on the shelf*	4.64 ± 10.92	3.60 ± 10.53	1.97 ± 4.91	7.49	0.0006	0.0348
Quadrupedal walking on floor	78.92 ± 72.94	68.36 ± 63.45	75.14 ± 72.27	7.23	0.0007	0.0406
Climbing	13.82 ± 23.13	15.77 ± 28.73	9.59 ± 16.56	6.02	0.0025	0.1450
Walking on the iron chain*	0.78 ± 5.49	0.06 ± 1.00	0.05 ± 0.61	11.86	0.0000	0.0000
Walking on the skylight	1.53 ± 6.19	3.52 ± 19.79	0.58 ± 2.98	8.20	0.0003	0.0174
Standing	13.69 ± 29.86	9.87 ± 24.33	16.86 ± 30.71	9.16	0.0001	0.0058
Scratching by hind leg	2.41 ± 4.36	4.27 ± 7.32	2.64 ± 5.12	31.56	0.0000	0.0000
Scratching by foreleg	77.60 ± 131.87	56.85 ± 97.74	67.63 ± 104.64	9.83	0.0001	0.0058
Licking hand	5.43 ± 21.08	5.796 ± 38.69	17.89 ± 81.39	10.81	0.0000	0.0000
Rubbing paw on floor	0.19 ± 1.48	0.10 ± 1.13	1.71 ± 8.56	32.50	0.0000	0.0000
Licking tail	0.35 ± 3.12	2.46 ± 22.59	0.29 ± 2.01	6.41	0.0017	0.0986

Data listed as means ± SDs.

Post hoc testing found that controls presented with significant differences from NOD and SID subjects, but there were no differences found between NOD and SID subjects.

^∧^Bonferroni correction used to reduce type I error for multiple comparisons.

*P < 0.05.

**Table 7:** Behavioral Effects of Ketamine Administration by Duration and Frequency

		Healthy controls		NOD subjects		SID subjects	
	Behavior	Pre-admin	Post-admin	Pre-admin	Post- admin	Pre-admin	Post-admin
**Duration**	Chewing	80.86 ± 149.22	64.47 ± 138.30	21.26 ± 76.56	7.01 ± 32.64	58.60 ± 171.75	15.48 ± 49.65
	Feeding while perched	150.88 ± 274.64	0.00 ± 0.00	199.96 ± 311.26	128.95 ± 213.42	227.95 ± 400.90	4.19 ± 58.48
	Hanging on window or door	33.23 ± 82.93	67.46 ± 133.37	340.49 ± 460.57	554.48 ± 740.97	10.25 ± 31.90	64.29 ± 164.83
**Frequency**	Feeding while sitting	2.27 ± 4.35	2.35 ± 4.61	0.18 ± 0.70	0.05 ± 0.34	1.91 ± 4.27	0.96 ± 1.96
	Chewing	1.97 ± 4.07	1.33 ± 2.68	0.66 ± 2.22	0.11 ± 0.56	1.32 ± 3.72	0.43 ± 1.12
	Watching company	23.9 ± 11.04	18.54 ± 10.77	14.98 ± 9.65	12.20 ± 9.23	23.99 ± 10.94	15.54 ± 9.00
	Scratching by foreleg	6.47 ± 5.43	3.94 ± 4.02	2.82 ± 2.94	2.07 ± 2.16	6.97 ± 6.18	3.11 ± 3.29

Data listed as means ± SDs.

**Table 8:** Key Metabolites Differentiating NOD Subjects and Healthy Controls in the PLS-DA Model†

Metabolite	Ret (min)	m/z	Fold-change† (NOD/CON)	*P*-value‡	VIP*
Urea	7.90	189	−0.09	0.04	1.78
L-leucine	8.44	232	−0.06	0.01	2.15
Serine	9.66	147	−0.09	0.05	1.71
L-threonine	10.02	160	−0.11	0.04	1.72
Butanoic acid	10.33	103	−0.16	0.01	2.21
Silanamine	10.62	218	0.21	0.02	1.89
Threitol	11.52	205	−0.05	0.04	1.76
L-methionine	11.64	250	−0.19	0.00	2.30
L-proline	12.78	216	−0.12	0.04	1.75
L-phenylalanine	12.94	266	−0.11	0.03	1.84
L-asparagine	13.46	218	−0.18	0.04	1.76
Monoamidomalonic acid	14.01	319	−0.11	0.03	1.86
2-keto-gluconic acid	14.65	292	−0.12	0.02	2.01
Arabinofuranose	14.69	217	−0.17	0.02	1.96
Phosphoric acid	14.74	299	−0.13	0.04	1.75
L-ornithine	15.06	174	−0.14	0.02	1.91
Citric acid	15.14	347	−0.20	0.03	1.80
1,4-butanediamine	15.38	174	−0.12	0.04	1.77
Benzamide	15.82	198	0.19	0.01	2.18
Fructose	15.92	364	−0.25	0.00	2.69
Sedoheptulose	15.95	205	0.10	0.03	1.84
Glucose	16.10	217	−0.04	0.03	1.80
Glycerol	16.19	208	0.27	0.04	1.75
Inositol	16.63	318	−0.25	0.01	2.17
Myo-inositol	17.90	73	−0.13	0.02	1.93
Turanose	23.34	361	−0.18	0.03	1.82
α-D-glucopyranoside	23.54	361	−0.17	0.04	1.77

^†^Positive values indicate higher levels in naturally-occurring depressed subjects (NOD) relative to healthy controls (CON); negative values indicate lower levels in NOD relative to CON.

^‡^*P*-values were calculated from the Student's t-test.

*Variable importance in the projection (VIP) was acquired from the PLS-DA model with a threshold of 1.0.
